# Plasma fibrin clot properties and cardiovascular mortality in patients with type 2 diabetes: a long‐term follow‐up study

**DOI:** 10.1186/s12933-021-01230-9

**Published:** 2021-02-18

**Authors:** Agata Hanna Bryk, Małgorzata Konieczyńska, Maciej Polak, Dariusz Plicner, Maciej Bochenek, Anetta Undas

**Affiliations:** 1 Department of Endocrinology, Jagiellonian University Medical College, Krakow, Poland; 2 John Paul II Hospital, Krakow, Poland; 3 Department of Epidemiology and Population Studies, Institute of Public Health, Faculty of Health Sciences, Jagiellonian University Medical College, Krakow, Poland; 4 Unit of Experimental Cardiology and Cardiac Surgery, Faculty of Medicine and Health Sciences, Andrzej Frycz Modrzewski Krakow University, Krakow, Poland; 5 Clinic of Cardiac Transplantation and Mechanical Circulatory Support, Department of Heart Diseases, Wroclaw Medical University, Wrocław, Poland; 6 Institute of Cardiology, Jagiellonian University Medical College, 80 Pradnicka St., 31-202 Krakow, Poland

**Keywords:** Type 2 diabetes, Fibrin clot, D-dimer, Cardiovascular mortality

## Abstract

**Background:**

Patients with type 2 diabetes mellitus (T2DM) are at high risk of cardiovascular mortality, but the mechanisms behind this remain unclear. Prothrombotic fibrin clot properties have been shown in T2DM and cardiovascular disease. We hypothesized that formation of denser clots, which are resistant to fibrinolysis, has a negative impact on cardiovascular mortality in T2DM.

**Methods:**

We studied 133 T2DM patients aged 43–83 years. Plasma fibrin clot turbidity, permeation, compaction, and efficiency of clot lysis using 3 assays including the determination of maximum concentration (D-D_max_) and rate of increase in D-dimer concentration (D-D_rate_) released during tissue plasminogen activator-induced degradation, were evaluated at the time of enrollment, along with thrombin generation and fibrinolytic proteins. During a median follow-up period of 72 months, cardiovascular mortality was recorded.

**Results:**

Cardiovascular deaths (n = 16, 12%) occurred more frequently in patients with increased D-D_max_ (> 4.26 mg/l, hazard ratio [HR] 5.43, 95% confidence interval [CI] 1.99–14.79), or decreased D-D_rate_ (< 0.07 mg/l/min, HR 2.97, 95% CI 1.07–8.23), or increased peak thrombin (> 283.5 nM, HR 5.65, 95% CI 2.07–15.51). These predictors had an even more potent impact on cardiovascular mortality in patients with prior cardiovascular disease (64.7%) and with corresponding risks as follows: HR 6.18, 95% CI 2.02–18.96; HR 8.98, 95% CI 2.99–26.96; and HR 5.35, 95% CI 1.62–17.72, respectively. Other investigated fibrin variables and fibrinolytic proteins did not associate with cardiovascular mortality. In multivariable analysis, cardiovascular mortality was predicted by D-D_max_ > 4.26 mg/l, age > 65 years, prior cardiovascular disease, and C-reactive protein > 3 mg/l.

**Conclusions:**

This study is the first to show that formation of denser fibrin clots resistant to fibrinolysis could be a risk factor for long-term cardiovascular mortality in T2DM.

## Background

Type 2 diabetes mellitus (T2DM) is considered to be an epidemic of the twenty-first century, having a global prevalence of 9.3% in 2019 [1]. Over the last 15 years, the mortality and incidence of cardiovascular events has declined both in patients with and without T2DM, though at a slower rate among diabetic patients [2]. Cardiovascular disease affects 32.2% of T2DM patients and leads to mortality in 9.9% of patients having T2DM of more than 10 years duration [3]. Coronary artery disease (CAD) and stroke are major contributors to overall mortality in T2DM patients [3, 4].

There is growing evidence which shows that prothrombotic fibrin clot properties, including formation of more compact clots which are more resistant to lysis, are involved in atherothrombotic events such as myocardial infarction and ischemic stroke [5–7]. Several groups have also shown an association of T2DM with a prothrombotic clot phenotype [8–12]. Of note, T2DM has been reported to unfavorably affect plasma fibrin clot characteristics, such as leading to hypolysability, in patients with concomitant advanced CAD [13, 14]. The mechanisms behind impaired clot susceptibility to lysis observed in T2DM are complex. It has been suggested that hypofibrinolysis in T2DM is associated with elevated levels of plasminogen activator inhibitor-1 (PAI-1) [15] and thrombin-activatable fibrinolysis inhibitor (TAFI) [16], along with increased cross-linking of α2-antiplasmin into fibrin networks [8, 17], and glycation of fibrinogen [18] and plasminogen [19], while the mechanical properties of fibrin are independent from fibrinogen glycation [20]. Prolonged T2DM duration might also contribute to prothrombotic fibrin clot alterations including impaired fibrinolysis [12], with enhanced oxidative stress playing a substantial role [21]. Patient sex is also an important modulator of fibrin clot properties, both in T2DM and CAD patients [14, 22]. Asymptomatic women with coronary plaques, as visualized by coronary computed tomography angiography, have reduced fibrin clot lysability compared to men with and without coronary plaques, and women without coronary plaques, suggesting a sex-dependent link between coronary atherosclerosis and fibrin clot lysability [22].

Fibrinogen, the main component of fibrin, has been reported to be predictive of major adverse cardiovascular events, especially among diabetic patients, both following acute coronary syndrome [23] and in those with stable coronary disease [24]. Moreover, fibrin clots that are resistant to lysis have predicted adverse outcome in diabetic patients following acute coronary syndrome [25]. The same was true for maximum turbidity. In another study [26], area under the turbidity curve, but not the turbidity lysis time, associated with cardiovascular events in CAD patients, also after adjustment for T2DM, which was diagnosed in 29% of the study participants. To the best of our knowledge, there have not been any published reports investigating the long-term impact of fibrin clot phenotype on mortality in T2DM patients. We hypothesized that formation of denser clots, which are more resistant to fibrinolysis, leads to increased long-term cardiovascular mortality in T2DM.

## Methods

### Patients

Patients were recruited from March 2011 to November 2011 from the John Paul II Hospital, Krakow, Poland. The study population was previously described in detail [12]. Briefly, 156 patients with T2DM aged 18 years or greater who fulfilled the American Diabetes criteria for diagnosis of T2DM were enrolled. Patients were excluded if they presented with signs of acute infection, suffered from arterial or venous thromboembolic events within the six months prior to enrollment, were treated with anticoagulants at the time of enrollment, or were diagnosed with cancer, chronic inflammatory disease, liver injury, or stage 4 or 5 chronic kidney disease. Pregnant women were also excluded.

Data collected in the questionnaire included demographic data, time since T2DM diagnosis, comorbidities, and medications used. Obesity was defined as body-mass index > 30 kg/m^2^. Hypercholesterolemia was defined as low-density lipoprotein (LDL) cholesterol > 2.5 mmol/l or ongoing lipid-lowering treatment [27]. Other comorbidities were defined as previously described [12]. The study was approved by the local bioethics committee. All patients provided informed written consent.

### Laboratory investigations

At baseline, fasting blood samples were obtained from the antecubital vein between 8:00 and 10:00 am. Glycated hemoglobin (HbA1c), fasting glucose, creatinine, lipid profile, and high sensitivity C-reactive protein (CRP) were assayed by routine laboratory techniques. Fibrinogen was determined with the von Clauss method. Plasminogen and α2-antiplasmin were measured using chromogenic assays (STA Stachrom α2-antiplasmin and plasminogen, Diagnostica Stago, Asnieres, France). Commercially available immunoenzymatic assays were used to measure tissue plasminogen activator (tPA) antigen (TintElize, Umea, Sweden), PAI-1 antigen (American Diagnostica, Stamford, CT, USA), TAFI antigen (Chromogenix, Lexington, MA, USA), and soluble thrombomodulin (Diagnostica Stago).

Plasma thrombogenic potential was assessed using calibrated automated thrombography (Thrombinoscope BV, Maastricht, the Netherlands) in a 96-well plate fluorometer (Ascent Reader, Thermolab systems OY, Helsinki, Finland) at 37 °C according to the manufacturer’s instructions. A volume of 80 µl of platelet-poor plasma was diluted with 20 µl of a commercially available tissue factor-based activator (Diagnostica Stago) containing 5 pmol/l recombinant tissue factor, 4 µmol/l phosphatidylserine/phosphatidylcholine/phosphatidylethanolamine vesicles, and 20 µl of FluCa solution (Hepes, pH 7.35, 100 nmol/l CaCl_2_, 60 mg/ml bovine albumin, and 2.5 mmol/l Z-Gly-Gly-Arg-amidometylcoumarin). The peak thrombin level was analysed [28]. Plasma samples were analyzed in duplicate, and the intra-assay variability was 6%.

Fibrin clot parameters, assayed at the time of enrollment, included: turbidity, permeation, compaction, lysability, maximum concentration (D-D_max_), and rate of D-dimer (D-D_rate_) released during tPA-induced clot lysis, and were previously described in detail [12]. Two parameters were assayed during turbidity measurements: lag phase, which reflects the time required for fibrin protofibrils to grow to a sufficient length to allow for lateral aggregation, and maximum absorbance at plateau (ΔAbs), which reflects fiber cross-sectional area. Plasma was diluted 2:3 with 0.05 mol/l Tris buffer and was incubated with 1 U/ml human thrombin (Sigma) and 16 mmol/l calcium chloride. Absorbency was read every 7 s at 350 nm for 15 min with a Dynex MRX2 plate reader. Lag phase was recorded as the time needed for absorbency to change by 0.01 from baseline [29]. Fibrin clot permeation was determined by measuring flow rates of buffer through the fibrin clots obtained from the 120 µl of citrated plasma, by adding 20 mmol/l calcium chloride and 1 U/ml human thrombin, as previously described [30, 31]. The mass of consecutive drops through each tube during a period of 60 min was recorded for exact volume. A permeation coefficient (K_s_), indicating the average size of fibrin clot pores, was calculated from the following equation: K_s_ = $\frac{{\text{Q}}\times {\text{L}}\times\upeta }{{\text{t}}\times {\text{A}}\times \Delta {\text{p}}}$, where Q is the flow rate in time (t); L is the length of a fibrin gel; η is the viscosity of liquid (in poise); A is the cross-sectional area (in square centimeters), and Δp is the differential pressure (in dynes per square centimeter). To assess compaction, the citrated plasma was mixed (3:2) with 0.7 IU/ml thrombin, 0.1% Tween 80, and 20 mmol/l calcium chloride in TBS, and then clots were formed in tubes prepared as in the permeation experiments [31, 32]. After centrifugation at 6000 g for 60 s, the volume of supernatant evacuated from the tubes was assessed based on the difference in mass of the tube. Compaction was expressed as this volume divided by the initial plasma volume used to form the fibrin clot. Two plasma clot lysis assays were performed. In the modified assay by Lisman et al. [33], citrated plasma was mixed (1:1) with HEPES buffer containing calcium chloride (final concentration, 17 mmol/l), diluted recombinant tissue factor (final dilution 10^5^ times; Innovin, Dade Behring), phospholipid vesicles (final concentration, 10 µmol/l), and recombinant tPA (rtPA, final concentration, 30 U/mL, 56 ng/mL; Boehringer Ingelheim, Ingelheim, Germany). Clot lysis time (CLT) was defined as time from the midpoint of the baseline to maximum turbid transition, to the final plateau phase. In the second assay, using the modified approach by Williams et al. [34], 1 µg/ml rtPA (Boehringer Ingelheim) was added simultaneously with human thrombin. This lysis time was defined as the time required for a 50% decrease in clot turbidity measured at 405 nm (t_50%_) at 37 °C in a Spectra Max 340 kinetic microplate reader (Molecular Devices, Sunnyvale, CA, USA). Finally, plasma lysis was assessed using a modified version of the method described by Collet et al. [35, 36]. The plasma samples were recalcified with calcium chloride (final concentration, 20 mmol/l) and then 1 U/ml human thrombin (Sigma-Aldrich, St Louis, MO, USA) was added. After 2 h of incubation at room temperature, tubes containing the clots were connected via plastic tubing to a reservoir with buffer (0.05 M Tris–HCl, 0.15 M NaCl, pH 7.5), containing 0.2 µM rtPA. The lysis rate was determined by measuring the concentration of released D-dimers (American Diagnostica), a marker of plasmin-mediated fibrin degradation, in the eluate every 20 min. Two measures of fibrin clot architecture, namely the high maximum concentration of D-dimer concentration (D-D_max_) and the rate of increase in D-dimer concentrations released from the clot (D-D_rate_) were determined in each subject. The experiment was usually stopped after 80–120 min when the fibrin gel collapsed under the pressure.

### Follow-up

The primary endpoint was cardiovascular death. The length of follow-up was censored at the time of death. Follow-up by telephone was conducted between July 2017 and January 2018. The family of the deceased patient was asked about the cause of death. In the case of incomplete medical record data or loss to clinical follow-up, patient status was assessed in March 2019 using the National Mortality Registry maintained by the State Systems Department of Ministry of Digital Affairs. The International Statistical Classification of Diseases 10 (ICD-10) codes I70.9, I25.0, I25.9, and I11.0 were classified to be cardiovascular death. In the case of ICD-10 code R99, the correct classification of death was based on additional clinical data. The secondary endpoint was all-cause death.

### Statistical analysis

Continuous data were presented as medians (interquartile range, IQR) and were compared using the Mann–Whitney U test. Categorial data were presented as numbers (percentages). To test the differences in proportions between two groups, Fisher’s exact test was used. Continuous variables (peak thrombin, ΔAbs_max_, K_s_, compaction, CLT, t_50%_, D-D_max_, and D-D_rate_) were dichotomized using the cut-off values found in the analysis of receiver operating curves (ROC), which optimally classified the clinical endpoint, as described previously [37]. Multivariable Cox proportional hazards survival regression, after adjustment for age and sex, was used to investigate the effect of high peak thrombin, high ΔAbs_max_, low K_s_, high compaction, prolonged CLT, prolonged t_50%_, high D-D_max_, and low D-D_rate_, on clinical outcomes. The results were presented as hazard ratios (HR) with 95% confidence interval (CI). The HR were additionally adjusted for possible confounding factors, identified in the comparison analysis of two groups. Kaplan–Meier survival curves were drawn. Six factors (age > 65 years, previous cardiovascular event, CRP level > 3 mg/l, hypercholesterolemia defined as LDL > 1.8 mmol/l or treatment with statins, D-D_max_ and peak thrombin) were analyzed for the association with the cardiovascular mortality in the univariate logistic regression model. The Nagelkerke R^2^ represented the contribution of D-D_max_ and peak thrombin to the variation in cardiovascular mortality. Factors, which significantly associated with cardiovascular mortality in the univariate regression model, were included in the multivariable logistic regression model. Results of the regression model were presented as odds ratio (OR) with 95% CI. To address the problem of multiple comparisons, the results were controlled with the Benjamini–Hochberg procedure. If continuous variables correlated with Spearman’s correlation coefficient > 0.5, only one of them was included in the multivariable regression model. A two-sided p-value of < 0.05 was considered statistically significant. No formal sample size calculation was performed, as the number of patients included in the original study was fixed [12]. We performed a post hoc power calculation based on a 12% event rate, HR of 5.4, with the distribution of patients in the subgroups 20%/80%. Given these estimates, and with a significance level set at 5%, a post hoc power of 78% was found for D-D_max_. Statistical analyses were performed using IBM SPSS Statistics for Windows, Version 25.0. (Armonk, NY: IBM Corp.). The Benjamini–Hochberg procedure was applied in R [38]. Figures were prepared using GraphPad Prism (version 8.4.2 for Windows, GraphPad Software, San Diego, CA, USA).

## Results

### Patient characteristics at baseline

A total of 156 patients with T2DM (87 men and 69 women) were enrolled. At the time of enrollment, median period since diagnosis of T2DM was 5.0 (IQR, 2.0–10.0) years, while the HbA1C levels ranged from 5.2 to 12.6 (median, 6.5) %. The patients were mostly (60.3%) treated with biguanides, while 26.3% of patients received insulin. A history of cardiovascular events was recorded in 67.3% of patients, including 64.1% with CAD.

### Patient characteristics during follow-up with respect to cardiovascular death in the entire study group

The median length of follow-up was 72 (68–74) months. Twenty-three (14.7%) patients were lost to follow-up. Final analysis involved 133 patients, 79 men and 54 women, aged 43.0–83.0 (median 66.0) years. During follow-up, 16 patients (12.0%) died from cardiovascular causes (2.18 per 100 patient-years). Patients who died from cardiovascular causes did not differ from the survivors in terms of demographic data and time since T2DM diagnosis, but they more commonly had nephropathy at baseline and less commonly were treated with metformin when compared to the survivors (Table 1). Despite similar HbA1c, patients who died from cardiovascular causes tended to have lower fasting glucose levels when compared with the remaining patients (Table 2). Patients who died from cardiovascular causes had lower total and LDL-cholesterol and higher CRP when compared with the remaining patients (Table 2). Patients with LDL-cholesterol above the a median, i.e. ≥ 2.3 mmol/l, did not differ from the remaining subjects in regard to the parameters of clot structure and function. The baseline fibrinogen concentration, tPA and PAI-1 antigen, α2-antiplasmin and TAFI activity, as well as thrombomodulin antigen did not differ between patients who died from cardiovascular causes and the survivors (Table 2). Analysis of several variables describing plasma fibrin clot structure and function showed that the clot turbidity, permeability, compaction, and lysability, represented by CLT, were comparable in patients who died from cardiovascular causes and the survivors (Table 2). Patients who died from cardiovascular causes had higher D-D_max_ and lower D-D_rate_ when compared with the survivors (Table 2). The absolute difference in D-D_rate_ between those groups corresponds with the standardized effect size of 0.68, and it is statistically significant in the Mann–Whitney’s test.

**Table 1:** Baseline characteristics of type 2 diabetes mellitus (T2DM) patients divided according to the cardiovascular death

Variable	Non-survivors (n = 16)	Survivors (n = 117)	p-value
Age, years	70.5 (64.0–77.0)	65.0 (60.0–71.0)	0.09
Female gender, n (%)	5 (31.3)	49 (41.9)	0.59
Current smoking, n (%)	2 (12.5)	10 (8.6)	0.64
Body-mass index, kg/m^2^	31.4 (26.2–34.2)	31.6 (28.4–35.4)	0.34
Obesity, n (%)	10 (62.5)	80 (68.4)	0.78
Time since T2DM diagnosis, years	8.0 (2.0–13.0)	5.0 (2.0–10.0)	0.43
Comorbidities, n (%)			
Arterial hypertension	14 (87.5)	112 (95.7)	0.20
Coronary artery disease	13 (62.3)	73 (62.4)	0.17
Previous myocardial infarction	6 (37.5)	19 (16.2)	0.08
Previous stroke	3 (18.8)	4 (3.4)	0.04
Nephropathy	6 (37.5)	17 (14.5)	0.03
Neuropathy	4 (25.0)	20 (17.1)	0.49
Retinopathy	4 (25.0)	17 (14.5)	0.28
Medications, n (%)			
Acetylsalicylic acid	13 (81.3)	92 (78.6)	1.00
Statin	13 (81.3)	93 (79.5)	1.00
ACE-I	10 (62.5)	81 (69.2)	0.58
β-blocker	14 (87.5)	91 (77.8)	0.52
Metformin	5 (31.3)	75 (64.1)	0.02
Sulfonylourea	9 (56.3)	47 (40.2)	0.28
Insulin	6 (37.5)	27 (23.1)	0.23

Continuous data are presented as the median (interquartile range) and compared using Mann–Whitney test. Categorical data are presented as number (percentages) and compared using Fisher’s exact test

*ACEI* angiotensin converting enzyme inhibitor

**Table 2:** Baseline laboratory investigations of type 2 diabetes mellitus patients divided according to the cardiovascular death

Variable	Non-survivors (n = 16)	Survivors (n = 117)	P-value
Basic			
HbA1c, %	6.7 (6.2–8.5)	6.4 (6.1–7.1)	0.25
Fasting glucose, mmol/l	5.0 (4.2–6.5)	6.0 (5.3–7.4)	0.06
Creatinine, µmol/l	82.0 (77.0–113.5)	81.0 (65.0–96.0)	0.17
Total cholesterol, mmol/l	3.4 (3.3–4.4)	4.3 (3.6–5.0)	0.03
LDL-cholesterol, mmol/l	1.7 (1.4–2.4)	2.4 (1.9–3.0)	0.01
HDL-cholesterol, mmol/l	1.2 (1.0–1.5)	1.3 (1.1–1.6)	0.30
Triglycerides, mmol/l	1.0 (0.9–1.6)	1.3 (1.0–1.8)	0.22
High-sensitivity C-reactive protein, mg/l	3.3 (1.9–6.0)	1.9 (1.0–3.4)	0.04
Coagulation parameters			
Fibrinogen, g/l	3.2 (2.8–3.5)	3.0 (2.6–3.5)	0.32
Peak thrombin, nM	253.5 (212.0–289.5)	228.0 (201.0–268.0)	0.17
Plasminogen activity, %	105.5 (88.5–115.5)	105.0 (98.0–117.0)	0.48
tPA antigen, ng/ml	11.8 (9.3–15.6)	11.2 (10.0–13.2)	0.49
PAI-1 antigen, ng/ml	32.1 (29.5–37.1)	32.1 (29.5–37.1)	0.88
α2-antiplasmin activity, %	115.0 (101.0–121.0)	115.0 (101.0–121.0)	0.16
TAFI activity, %	97.5 (89.0–106.0)	97.5 (89.0–106.0)	0.72
Thrombomodulin antigen, ng/ml	3.1 (2.3–3.6)	3.01 (2.3–3.6)	0.91
Fibrin clot properties			
Lag phase, s	42.5 (40.0–44.5)	43.0 (40.0–46.0)	0.67
∆Abs, 405 nm	0.825 (0.800–0.860)	0.810 (0.770–0.850)	0.26
K_s_, × 10^–9^ cm^2^	7.0 (6.2–7.3)	7.1 (6.6–7.7)	0.13
Compaction, %	44.0 (39.5–46.0)	44.0 (40.0–49.0)	0.47
CLT, min	88.0 (79.0–104.5)	95.0 (82.0–105.0)	0.45
t_50%_, min	10.15 (9.60–10.95)	9.8 (9.0–10.4)	0.06
D-D_max_, mg/l	4.18 (3.77–4.48)	3.85 (3.60–4.09)	0.01
D-D_rate_, mg/l/min	0.069 (0.064–0.071)	0.070 (0.068–0.073)	0.02

Data are presented as the median (interquartile range) and compared using Mann–Whitney test

*HbA1c* glycated hemoglobin, *LDL* low-density lipoprotein, *HDL* high-density lipoprotein, *tPA* tissue plasminogen activator, *PAI* plasminogen activator inhibitor 1, *TAFI* thrombin-activatable fibrinolysis inhibitor, *K*_*s*_ permeability coefficient, *∆Abs* maximum absorbance at plateau, *t*_*50%*_ the time required for a 50% decrease in clot turbidity, *CLT* clot lysis time, *D-D*_*max*_ maximum D-dimer concentrations, *D-D*_*rate*_ maximum rates of increase in D-dimer levels

### Correlations between the investigated parameters in the entire study group

The D-D_max_ correlated with ∆Abs (r = 0.432, p < 0.001), K_s_ (r = − 0.496, p < 0.001), compaction (r = − 0.446, p < 0.001), CLT (r = − 0.463, p < 0.001), and t_50%_ (r = 0.518, p < 0.001), but not with peak thrombin (r = 0.14, p = 0.10). The correlations between D-D_max_ and CRP, and between D-D_rate_ and CRP, were weak (r = 0.208, p = 0.020, and r = − 0.208, p = 0.020, respectively).

### Predictors of cardiovascular mortality in the entire study group

**Fig. 1:**
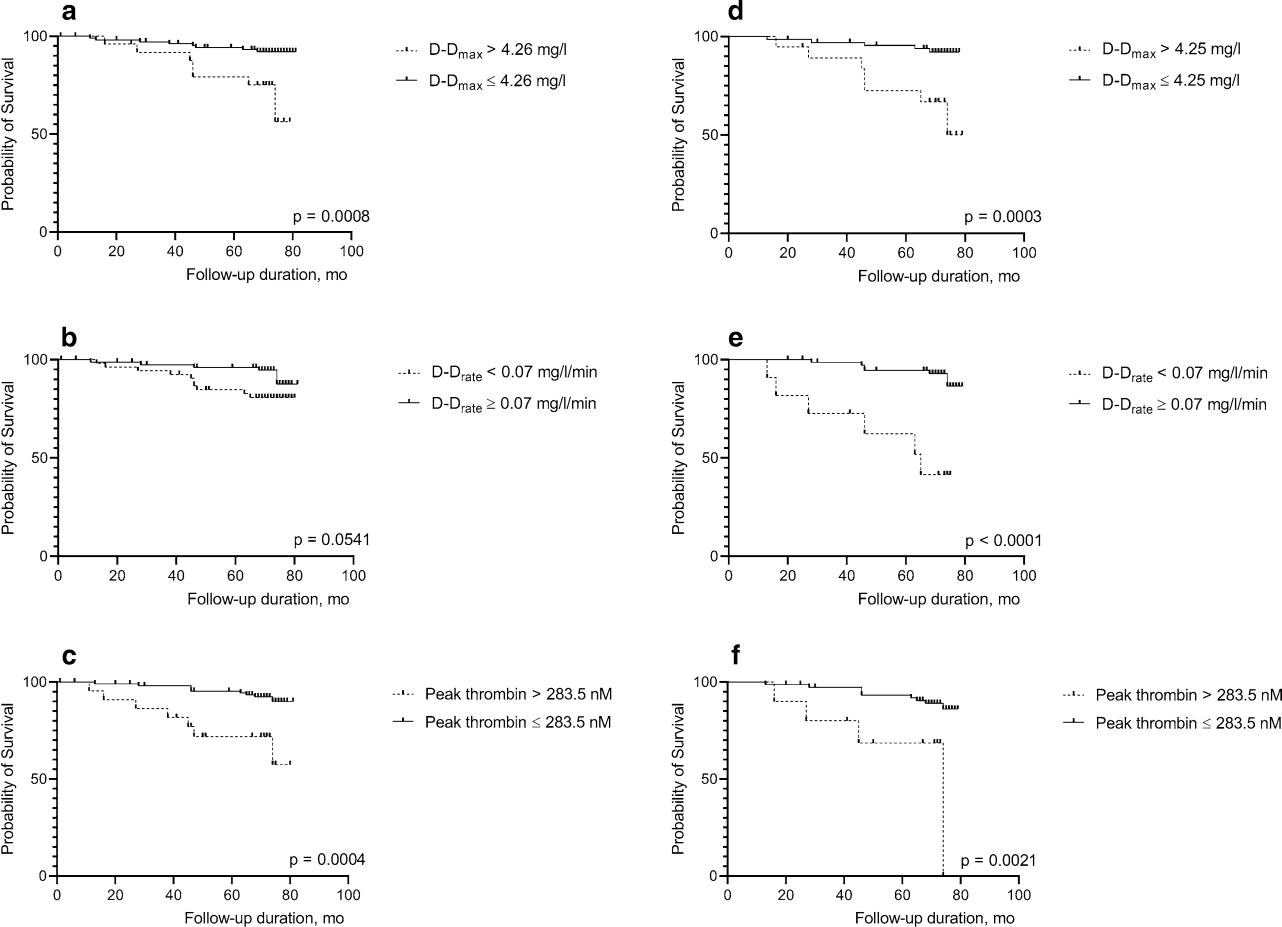
Kaplan–Meier curves showing probability of survival in diabetic patients (**a**–**c**) and in diabetic patients with cardiovascular disease (**d**–**f**) with increased maximum D-dimer concentration (D-D_max_), or decreased rate of D-dimer (D-D_rate_) released during clot lysis, or increased peak thrombin generated, as compared with the remainder. Cut-offs were estimated based on the receiver operating curves. The D-D_max_ > 4.26 mg/l was regarded as increased in all diabetic patients, while D-D_max_ > 4.25 mg/l was regarded as increased in diabetic patients with cardiovascular disease; in both groups D-D_rate_ < 0.07 mg/l/min was considered to be decreased, and peak thrombin > 283.5 nM was regarded as increased. P-values were calculated in the Mantel-Cox test

**Fig. 2:**
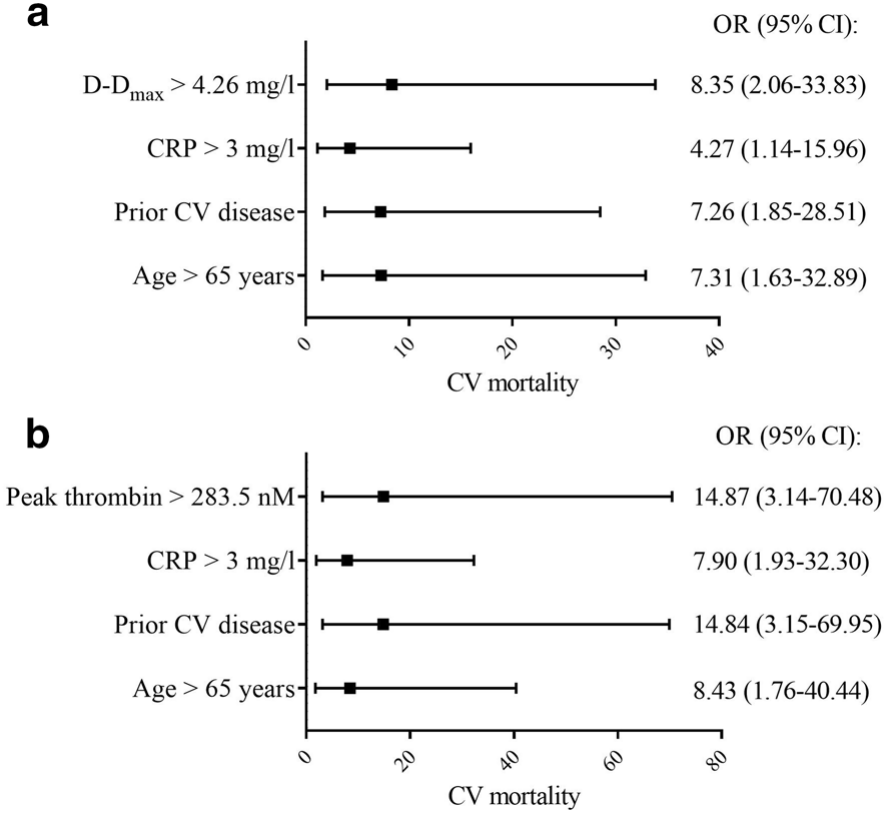
Associations of increased maximum concentration of D-dimer released during clot lysis (D-D_max_), peak thrombin and cardiovascular risk factors with cardiovascular mortality. Cardiovascular risk factors included: age > 65 years, prior cardiovascular disease, C-reactive protein > 3 mg/l. The D-D_max_ and peak thrombin were dichotomized using the cut-off value found in the receiver operating curves which optimally classified the cardiovascular death. A D-D_max_ > 4.26 mg/l and peak thrombin > 283.5 nM were regarded as increased. Squares represent odds ratio (OR) estimates, and horizontal lines represent 95% confidence intervals (CI), found in the multivariable regression analysis. CV denotes cardiovascular; CRP, C-reactive protein

Based on the ROC curves, D-D_max_ > 4.26 mg/l was regarded as a decreased value and D-D_rate_ < 0.07 mg/l/min was considered as decreased. At baseline, increased D-D_max_ was noted in 25 patients (18.8%), while decreased D-D_rate_ was observed in 54 (40.6%) patients. Cardiovascular deaths occurred more frequently in patients with increased maximum concentration of D-dimer released from the clot during lysis induced by tPA (D-D_max_) and decreased rate of D-dimer release (D-D_rate_, Table 3, Fig. 1a, b). Another predictor of cardiovascular death was increased peak thrombin (Table 3, Fig. 1c). Based on the ROC curves, increased peak thrombin > 283.5 nM was noted in 23 (17.3%) patients at baseline. Adjustment for cardiovascular disease history prior to the study enrollment, nephropathy, or treatment with metformin did not influence the hazard ratios (Table 3). In univariate regression model analysis, the high D-D_max_ was responsible for 13% variation in cardiovascular mortality (OR 5.88, 95% CI 1.94–17.79), while high peak thrombin accounting for 10% of that variation (OR 4.91, 95% CI 1.60–15.04). Since D-D_rate_ correlated with D-D_max_ (r = − 0.536, p < 0.001), only D-D_max_ was included in the regression model. The multivariable model including increased D-D_max_, along with traditional risk factors for cardiovascular mortality such as age > 65 years, cardiovascular events prior to study enrollment (myocardial infarction or stroke), and inflammatory state represented by CRP > 3 mg/l, accounted for 37% variation in cardiovascular death (Fig. 2a). When increased D-D_max_ was replaced in the model by increased peak thrombin, the explained variation in cardiovascular mortality was 42% (Fig. 2b). The Benjamini–Hochberg procedure controls did not affect results obtained in the multivatiate regression model. Despite the fact that D-D_max_ correlated with many of the investigated parameters as presented above, only D-D_max_ was found to be a predictor of cardiovascular death.

**Table 3:** Predictors of cardiovascular and all-cause mortality in type 2 diabetes mellitus patients

Predictor	HR* (95% CI)	HR** (95% CI)	HR*** (95% CI)	HR**** (95% CI)
Cardiovascular mortality				
D-D_max_ > 4.26 mg/l	5.43 (1.99–14.79)	5.79 (2.08–16.16)	5.08 (1.85–13.9)	6.85 (1.85–13.90)
D-D_rate_ < 0.07 mg/l/min	2.97 (1.07–8.23)	4.52 (1.47–13.86)	3.49 (1.24–9.82)	3.77 (1.33–10.69)
Peak thrombin > 283.5 nM	5.65 (2.07–15.51)	9.23 (2.97–28.86)	6.50 (2.34–18.18)	7.10 (2.52–20.05)
All-cause mortality				
D-D_max_ > 4.26 mg/l	3.48 (1.45–8.39)	3.61 (1.49–8.77)	3.45 (1.43–8.30)	3.85 (1.57–9.44)
D-D_rate_ < 0.07 mg/l/min	3.29 (1.31–8.28)	3.97 (1.50–10.46)	3.28 (1.03–8.24)	3.06 (1.18–7.90)
Peak thrombin > 283.5 nM	6.54 (2.78–15.35)	9.66 (3.72–25.09)	6.69 (2.85–15.69)	7.91 (3.28–19.07)

D-D_max_, D-D_rate_ and peak thrombin were dichotomized using the cut-off value found in the receiver operating curves that optimally classified the cardiovascular and all-cause death. Data were presented as hazard ratio (HR) with 95% confidence interval (CI) calculated in multivariable Cox proportional hazards survival regression

*D-D*_*max*_ maximum concentration of D-dimer concentration released from the clot during lysis induced by tissue plasminogen activator, *D-D*_*rate*_ maximum rates of increase in D-dimer levels

Hazard ratios adjusted for sex and age (*); for sex, age and cardiovascular disease history (**); for sex, age and nephropathy (***); for sex, age and metformin (****)

### Predictors of cardiovascular mortality in the subgroup of patients with prior cardiovascular disease

When T2DM patients with a history of cardiovascular disease prior to enrollment were analyzed separately (n = 86, 64.7%), 13 (15.1%) deaths were recorded. The predictors of cardiovascular mortality were D-D_max_ (HR 6.18, 95% CI 2.02–18.96, Fig. 1d) and D-D_rate_ (HR 8.98, 95% CI 2.99–26.96, Fig. 1e), as well as peak thrombin (HR 5.35, 95% CI 1.62–17.72, Fig. 1f).

### Predictors of all-cause mortality in the entire study group

During follow-up, all-cause death was recorded in 22 patients (16.5%, 2.99 per 100 patient-years), indicating 6 non-cardiovascular fatal events. Non-survivors were older (72.0 [66.0–77.0] vs. 65.0 [59.0–71.0] years, p = 0.004) when compared with survivors. Other demographic data, time since T2DM diagnosis, comorbidities, medications, and laboratory investigations, including fibrin properties, did not differ between the two groups. Similar to cardiovascular mortality, Kaplan–Meier curves showed that all-cause mortality was predicted by high D-D_max_, low D-D_rate,_ and high peak thrombin with similar cut-offs and HR with 95% CI as for cardiovascular mortality, before and after adjustment for sex, age, cardiovascular disease history, nephropathy, and metformin treatment (Table 3).

### Comparison of patients who died from non-cardiovascular vs. cardiovascular causes

When compared with patients who died from cardiovascular causes (n = 16, 12%), patients who died from non-cardiovascular causes (n = 6, 4.5%) were less frequently treated with β-blockers (2 [33.3] vs. 14 [87.5], p = 0.03) and had a higher concentration of LDL-cholesterol by 0.9 mmol/l (p = 0.03), although the frequency of treatment with statin was comparable between groups. Moreover, patients who died from non-cardiovascular causes had 11.4% increased clot compaction (p = 0.008), 7.4% shorter t_50%_ (p = 0.049), and tended to have lower D-D_max_ (p = 0.08) when compared with patients who died from cardiovascular causes.

## Discussion

To the best of our knowledge, this study is the first to evaluate the association between plasma fibrin clot properties and cardiovascular mortality during long-term follow-up of T2DM patients. Among several fibrin variables tested at enrollment, only D-D_max_ and D-D_rate_ were identified to be independent predictors of cardiovascular mortality in T2DM patients. These fibrin-related parameters (not associated with plasma fibrinogen concentration), in addition to advanced age, prior cardiovascular disease, and elevated CRP levels, helped to identify the risk of cardiovascular death. These associations were even more pronounced when patients with concomitant T2DM and prior cardiovascular disease were analyzed.

### Assay utilizing D-dimer concentrations: clinical relevance, possible mechanisms, and perspectives for future studies

Two measures of fibrin clot architecture determined in the assay introduced by Collet et al. in 1999 [35] indicate slightly different features of the clot. The D-D_max_ indicates the fibrin mass subjected to lysis by rtPA, while the D-D_rate_ reflects the ability of rtPA to penetrate into the fibrin clots. To understand the clinical relevance of these two parameters, it should be clear that opposite results associate with unfavorable fibrin clot characteristics: increased D-D_max_ and decreased D-D_rate_. In contrast to turbidity, where fibrin clot formation and degradation take place simultaneously [39], D-D_rate_ is the measure of tPA-induced degradation of the previously prepared plasma clots, generated in the presence of thrombin and calcium. The assay in which D-D_rate_ or D-D_max_ are evaluated may reflect the situation in which accumulating intravascular fibrin is exposed to exogenous tPA. For example, damage to the atherosclerotic plaque, which is rich in fibrin [40], results in the formation of fibrin on the surface of the exposed site [41]. A modified version of the method described by Collet et al. [35, 36] might be considered as a method to measure fibrin removal from the atherosclerotic plaques. We observed the association of D-D_rate_ and/or D-D_max_ with cardiovascular events also in our previous studies [36, 42]. Cardiovascular mortality was not predicted by other fibrinolytic tests (CLT and t_50%_) in which much lower concentrations of rtPA were applied, similar to those encountered during thrombolysis [43]. Results from our study support the use of a comprehensive set of plasma fibrin clot assays over the single test to better characterize fibrin clots in a given clinical setting.

The mechanisms behind impaired fibrinolysis in T2DM patients, including non-survivors during long-term follow-up, remain unclear despite extensive experimental efforts over the last decade [44]. Adjustment for potential clinical confounders, such as metformin treatment [45, 46], did not influence the results. Higher cardiovascular mortality was not attributed to the differences in fibrinogen concentration, the most important modulator of fibrin clot properties [8, 47], or PAI-1, or TAFI. It is hypothesized that post-translational modifications of fibrinogen (i.e. glycation and oxidation) are involved [48, 49].

Our study opens the discussion about the fibrin-targeted pharmacotherapy, which could improve the clinical outcomes in T2DM patients. Antithrombotic agents, such as aspirin monotherapy or in combination with low-dose rivaroxaban, have a favorable impact on cardiovascular mortality in T2DM [50]. The same agents can improve fibrin clot properties, including the acceleration of fibrinolysis [51, 52]. Prevention of mortality among T2DM patients receiving antithrombotic medications might be partly attributed to improved fibrin clot phenotype. Larger studies, including patients receiving antithrombotic agents, are needed to validate this intriguing hypothesis. Furthermore, it remains unclear whether the cardiovascular protective role of sodium-glucose cotransporter inhibitors or glucagon-like peptide-1 receptor agonists in T2DM is related to an improvement in fibrin clot properties.

### Other predictors of cardiovascular death: thrombin generation and CRP

Thrombin generation, as a potential predictor of cardiovascular mortality in T2DM, deserves a comment, given the contradictory results between the Ludwigshafen Risk and Cardiovascular Health (LURIC) [53] and Gutenberg Health Studies [54]. Our study provides data in favor of the positive association of thrombin generation and cardiovascular mortality in T2DM. Elevated CRP was another predictor of cardiovascular death in our study. Previously, elevated CRP level has been reported to be a predictor of cardiovascular and all-cause mortality in T2DM patients [55], although it has not been found to independently predict fibrin clot lysis in T2DM [9]. We speculate that elevated CRP increases the negative impact of dense fibrin clot formation, which are resistant to lysis in T2DM, although the mechanisms of this link remain elusive.

### Study limitations

Our study has several limitations. Firstly, the number of study participants was limited; however, the study was sufficiently powered to detect associations between plasma fibrin clot and cardiovascular events. The small sample size may have impeded adequate identification of possible confounding factors on the results. Non-survivors may have been a group with more advanced co-morbidities which could be reflected by a higher percentage of patients with nephropathy and prior stroke, when compared with survivors. Nevertheless, adjustment for previous cardiovascular events and nephropathy did not significantly influence the hazard ratios of investigated parameters for cardiovascular death. Larger studies in this area could allow for better distribution of comorbidities between the studied groups.

Secondly, antidiabetic medications being used and glycemic control, as reflected by HbA1c, were not assessed during follow-up. Because the median time since T2DM diagnosis at baseline was 5 years, we suspect that the impact of disease duration outweighed the effects of possible periods of inadequate glycemic control during that time [12].

Thirdly, use of the modified assay by Collet et al. in clinical practice is limited due to lack of standardization and the fact that it is a time-consuming protocol, requiring hands-on laboratory expertise to obtain reproducible results [35]. Our findings clearly show that further efforts to optimize the determination method of fibrin clot density and lysability in real-life patients are worthwhile as they might be clinically relevant in formulating a prognosis related to cardiovascular mortality. Another limitation of the study was that our analysis included only one of the thrombin generation parameters, i.e. peak thrombin. Although endogenous thrombin potential could add some extra information, both parameters are well correlated with each other [56].

Finally, we did not analyze specific cardiovascular events during follow-up since we focused on death as the single hard endpoint.

## Conclusions

In summary, our current study shows that the formation of denser clots, which are resistant to fibrinolysis, may predict cardiovascular mortality in T2DM.

## Data Availability

The datasets used and/or analyzed during the current study are available from the corresponding author on reasonable request.

## Abbreviations

ΔAbs: Maximum absorbance at plateau
CAD: Coronary artery disease
CI: Confidence interval
CLT: Clot lysis time
CRP: C-reactive protein
D-D_max_: Maximum concentration of D-dimer released during tissue plasminogen activator induced clot lysis
D-D_rate_: Rate of increase in D-dimer concentration released during tissue plasminogen activator-induced clot lysis
HbA1c: Glycated hemoglobin
HR: Hazard ratio
K_s_: Darcy’s constant or clot permeability
LDL: Low-density lipoprotein
OR: Odds ratio
PAI-1: Plasminogen activator inhibitor 1
ROC: Receiver operating curve
rtPA: Recombinant tissue plasminogen activator
t_50%_: Time required for a 50% decrease in clot turbidity
TAFI: Thrombin-activatable fibrinolysis inhibitor
tPA: Tissue plasminogen activator
